# Risk factors for tuberculous or nontuberculous spondylitis after percutaneous vertebroplasty or kyphoplasty in patients with osteoporotic vertebral compression fracture: A case-control study

**DOI:** 10.3389/fsurg.2022.962425

**Published:** 2022-08-18

**Authors:** Bo-Wen Zheng, Fu-Sheng Liu, Bo-Yv Zheng, Hua-Qing Niu, Jing Li, Guo-Hua Lv, Ming-Xiang Zou, Zhun Xu

**Affiliations:** ^1^ Department of Spine Surgery, The First Affiliated Hospital, Hengyang Medical School, University of South China, Hengyang, China; ^2^ Department of Spine Surgery, The Second Xiangya Hospital, Central South University, Changsha, China; ^3^ Musculoskeletal Tumor Center, Peking University People’s Hospital, Peking University, Beijing, China; ^4^ Department of Orthopedics Surgery, General Hospital of the Central Theater Command, Wuhan, China

**Keywords:** percutaneous vertebroplasty, percutaneous kyphoplasty, tuberculous spondylitis, nontuberculous spondylitis, pyogenic spondylitis, risk factors

## Abstract

**Objectives:**

The contributing factors for spondylitis after percutaneous vertebroplasty (PVP) or percutaneous kyphoplasty (PKP) remain unclear. Here, we sought to investigate the factors affecting spondylitis occurrence after PVP/PKP. We also compared the clinical characteristics between patients with tuberculous spondylitis (TS) and nontuberculous spondylitis (NTS) following vertebral augmentation.

**Methods:**

Literature searches (from January 1, 1982 to October 16, 2020) using MEDLINE, EMBASE, Google Scholar and Web of science databases were conducted to identify eligible studies according to predefined criteria. The local database was also retrospectively reviewed to include additional TS and NTS patients at our center.

**Results:**

Thirty studies from the literature and 11 patients from our local institute were identified, yielding a total of 23 TS patients and 50 NTS patients for analysis. Compared with NTS group, patients in the TS group were more likely to have a history of trauma before PVP/PKP treatment. Univariate analyses of risk factors revealed pulmonary tuberculosis and diabetes were significant factors for TS after PVP/PKP. Analyzing NTS, we found obesity, a history of preoperative trauma, urinary tract infection, diabetes and multiple surgical segments (≥2) were significantly associated with its occurrence following PVP/PKP treatment. Multivariate logistic analyses showed a history of pulmonary tuberculosis and diabetes were independent risk factors for TS after PVP/PKP, while diabetes and the number of surgically treated segments independently influenced NTS development.

**Conclusions:**

A history of pulmonary tuberculosis and diabetes were independent risk factors for TS. For NTS, diabetes and the number of surgically treated segments significantly influenced the occurrence of postoperative spinal infection. These data may be helpful for guiding risk stratification and preoperative prevention for patients, thereby reducing the incidence of vertebral osteomyelitis after PVP/PKP.

## Introduction

Percutaneous vertebroplasty (PVP) or percutaneous kyphoplasty (PKP) is currently widely used for the treatment of osteoporotic vertebral compression fractures (OVCFs) (1). Although it is relatively safe and effective, PVP/PKP can still cause complications in some situations. Among them, bone cement leakage is most frequently encountered and may lead to neurological dysfunction or even pulmonary embolism. Generally, infection of the vertebral body treated with subsequent PVP/PKP is rare, with an incidence of less than 1% (2). The most common type of spondylitis is purulent infection caused by bacteria (3). In addition, cases of tuberculous spondylitis (TS) after bone cement infusion have also been documented in the literature (2, 3). TS, the most common and severe form of bone tuberculosis, accounts for 50% of extrapulmonary tuberculosis cases and its incidence is very low in developed Western countries (4), while in developing countries, probably due to the lack of medical equipment (e.g., imaging systems and examination laboratories) and inadequate levels of diagnosis and treatment, the mortality rate from tuberculosis is much higher than in developed Western countries (4).

Currently, the cause of spondylitis after PVP/PKP remains unclear. Studies have demonstrated that the pathogen may already exist in patients before PVP/PKP treatment, the process of bone cement injection and vertebral augmentation initiates the occurrence of subsequent spinal infections (2). For example, infections involving the visceral organs (such as urinary tract infection, cholecystitis, meningitis) or pathogen adhesion in the skin may contribute to nontuberculous spondylitis (NTS) after PVP/PKP (2, 5, 6). Regarding TS following PVP/PKP surgery, some studies have proven that a history of pulmonary tuberculosis is closely related to the occurrence of spondylitis (2, 7, 8).This may be due to the presence of tuberculosis bacteria in recovered pulmonary tuberculosis patients, and PVP/PKP may allow these quiescent tuberculosis bacteria to spread around the bone cement, leading to infection (2).

Noticeably, patients undergoing PVP/PKP therapy generally have an advanced age, and infectious spondylitis in this patient group tends to progress rapidly once it develops (9), which may pose a challenge for subsequent treatment (usually requiring traumatic debridement surgery and long-term use of antibacterial drugs with side effects (2, 10, 11), and it can even lead to catastrophic consequences. Therefore, it is necessary to summarize the influencing factors of secondary vertebral infection after PVP/PKP to guide prevention approaches to reduce postoperative spinal infections, thus improving the clinical outcome of patients. In this study, we aimed to investigate the factors affecting spondylitis occurrence after PVP/PKP. We also compared the clinical characteristics between patients with TS and NTS.

## Methods and materials

### Literature review

**Figure 1:**
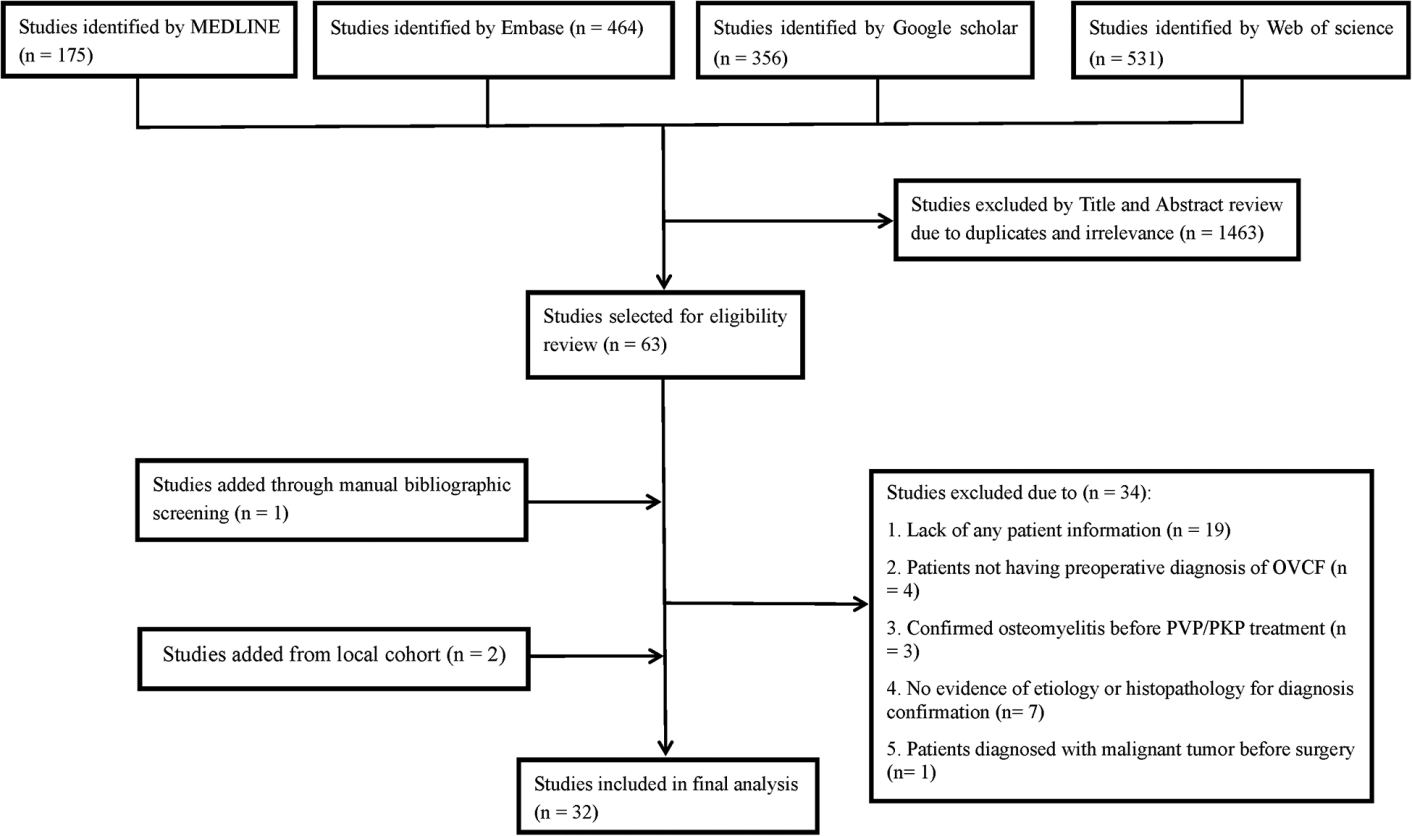
Flow diagram of literature search showing studies identified, included and excluded at each stage.

A literature search through the MEDLINE, EMBASE, Google Scholar and Web of science databases was conducted to identify eligible studies from January 1, 1982 to October 16, 2020. The keywords or combinations used for the search were (“spondylitis” or “spondylodiscitis” or “osteomyelitis” or “bacterial” or “fungal” or “pyogenic” or “tuberculosis” or “bacterial spondylitis” or “pyogenic spondylitis” or “tuberculous spondylitis” or “tubercular spondylitis” or “mycobacteria tuberculosis” or “TB” or “Pott’s” or “infection” or “infectious”) and (“spine” or “spinal” or “vertebral” or “cervical spine” or “thoracic spine “or “lumbar spine”) and (“VP” or “PVP” or “PKP” or “vertebroplasty” or “kyphoplasty” or “augmentation” or “percutaneous vertebroplasty” or “percutaneous kyphoplasty”). To obtain comprehensive results and to avoid omissions, no restrictions were applied for the above keywords. Moreover, we also manually reviewed the references of the included studies to find any potential documents that met the inclusion criteria. The detailed process for the literature search is shown in Figure 1. We included OVCF (It is directly described in the literature, and no specific inspection method is described) patients who developed a vertebral infection (including TS and NTS) after undergoing PVP/PKP surgery. The exclusion criteria of the study included: failing to offer any evidence of etiology or histopathology for diagnosis confirmation (for NTS, the diagnosis should be based on the pathogenic growth observed in the culture of infected tissues, while a diagnosis of TS requires detection of *Mycobacterium tuberculosis* in the tissue culture, or positive acid-fast staining or pathology findings showing caseous necrosis and/or granulomatous inflammation and/or multinucleated giant cells); patients with confirmed vertebral osteomyelitis before PVP/PKP treatment; patients not having a preoperative diagnosis of OVCF (including those with pathological fractures or others); patients diagnosed with malignant or benign tumors before surgery; and patients without any information eligible for analysis.

Two investigators independently screened the publications based on the inclusion criteria and extracted clinical data for each patient. Any dispute was resolved through consensus. Patient information obtained from the studies included the following: demographics (age and sex), clinical characteristics (including OVCF location, number of segments treated by PVP/PKP and preoperative neurological function, a history of trauma (the specific injury mechanism is not explained in detail, and the description only reflects the “trauma history”), the presence or absence of pulmonary tuberculosis, obesity, smoking, and other comorbidities [such as diabetes, rheumatoid arthritis, pneumonia, chronic obstructive pulmonary disease, urinary tract infection, and high blood pressure], radiological findings (the occurrence of paravertebral abscesses at first diagnosis of infection), microbiological results and laboratory tests (including the pathogens as well as WBC, ESR, CRP levels at the time of diagnosis), treatment (including revision surgery or not and the specific type of surgery), the time interval between PVP/PKP and the first diagnosis of spinal infection, follow-up time and clinical outcomes of the patients (recovery, limited mobility/assisted walking and death).

### Local cohort

A total of 1935 OVCF patients who were treated with PVP/PKP in our institute from March 2003 to March 2020 were identified. This duration of study was determined as the similar period in which the included cases were reported in the literature to allow for comparability. The medical records of the patients were reviewed retrospectively to include eligible cases with postoperative spondylitis. Patients in the local cohort were diagnosed with osteoporosis by bone density scans; all 8 patients included in this institution fell from low. The diagnosis of postoperative NTS was confirmed by microbiological evidence showing pathogenic growth in tissue culture. Postoperative TS was determined by acid-fast staining and the histopathological results of the lesion tissues. In total, 6 NTS cases and 5 TS cases after PVP/PKP were identified in our hospital. The overall incidence of spinal infection following PVP/PKP surgery was 0.57%. Among the 11 cases with postoperative spondylitis, two TS cases were previously described in our study (9). Using the PS matching plug-in of SPSS, 114 patients who did not develop vertebral osteomyelitis after PVP/PKP treatment in our hospital during the same period were randomly selected as the control group, and there was no significant difference in age or sex between it and the infected groups (control vs. TS: *t* = 0.828, *P* = 0.645 for age and *χ*^2^ = 0.550, *P* = 0.458 for sex; control *vs* NTS: *t* = 0.003, *P* = 0.994 for age and *χ*^2^ = 2.253, *P* = 0.133 for sex). The included patients with PVP/PKP in our hospital had normal preoperative inflammatory blood parameters and would not have undergone surgery otherwise. In addition, all patients received prophylactic intravenous antibiotics, specifically cefuroxime 0.5 g, on the day before surgery, the day of surgery, and the day after surgery. None of the patients included in our institution had any other form of surgical site infection or prolonged wound healing time during their hospitalization. Postoperatively, all patients underwent regular clinical and imaging follow-up, and the final follow-up time was November 2020. Patient clinical data were directly obtained from medical records.

### Statistical analyses

All statistical analyses were performed by using SPSS 26.0 (SPSS, Chicago, Illinois, USA). Continuous data are expressed as the mean ± standard deviation and were analyzed by Student's *t*-test, while categorical data are presented as the frequency or composition ratio and were analyzed by the chi-square test or Fisher's exact test. The multivariate logistic regression model was used to assess the independent risk factors for vertebral infection after PVP/PKP surgery, in which the factors that were found to be statistically significant (*P* < 0.1) in our univariate analysis, as well as important predictors reported in the literature, were included (2, 7, 8). All tests were two-sided, and *P* < 0.05 was considered to be statistically significant.

## Results

### Patient characteristics in the TS and NTS groups

A total of 30 studies met the inclusion criteria (2, 3, 5, 7–10, 12–34). Among them, 10 discussed TS after PVP/PKP, 19 analyzed the occurrence of postoperative NTS, and 1 evaluated both TS and NTS. After review, 20 TS patients and 44 NTS patients were identified from these studies. With an additional 5 TS patients and 6 NTS patients from our local center, a total of 23 TS patients and 50 NTS patients were finally included in this study. The clinical data of the included patients are shown in Tables 1, 2.

**Table 1:** Summary of the clinical characteristics in TS patients.

Variables	Categories	*n* (%)
Age (years)	Continuous	23 (71.5 ± 8.7)
Sex	Female	19 (82.6)
	Male	4 (17.4)
Preoperative neurological dysfunction	No	10 (90.9)
	Yes	1 (9.1)
Trauma	No	6 (42.9)
	Yes	8 (57.1)
Location	Thoracic	7 (30.4)
	Lumber	15 (65.2)
	Thoracic and Lumber	1 (4.4)
Number of surgically treated segments	One	17 (73.9)
	Two or more	6 (26.1)
Type of surgery	PVP	15 (65.2)
	PKP	8 (34.8)
Diabetes	No	15 (68.2)
	Yes	7 (31.8)
Rheumatoid arthritis	No	21 (95.5)
	Yes	1 (4.5)
Pulmonary tuberculosis	No	9 (45)
	Yes	11 (55)
COPD	No	20 (90.9)
	Yes	2 (9.1)
Hypertension	No	15 (68.2)
	Yes	7 (31.8)
WBC	Continuous	17 (7.4 ± 2.3)
ESR	Continuous	17 (52.4 ± 19.7)
CRP	Continuous	17 (42.7 ± 34.4)
Time interval to infection	Continuous	23 (8.5 ± 11.7)
Paravertebral abscess	No	1 (10)
	Yes	9 (90)
Outcomes	Recovery	11 (50)
	Death	3 (13.6)
	Walking assistance	8 (36.4)

TS, tuberculous spondylitis; PVP, percutaneous vertebroplasty; PKP, percutaneous kyphoplasty; COPD, chronic obstructive pulmonary diseases; WBC, white blood cell; ESR, erythrocyte sedimentation rate; CRP, C-reactive protein.

**Table 2:** Summary of the clinical characteristics in NTS patients.

Variable	Categories	*n* (%)
Age (years)	Continuous	50 (70.5 ± 10.4)
Sex	Female	32 (64)
	Male	18 (36)
Preoperative neurological dysfunction	No	11 (100)
	Yes	0 (0)
Trauma	No	21 (80.8)
	Yes	5 (19.2)
Location	Thoracic	15 (30.6)
	Lumber	31 (63.3)
	Thoracic and Lumber	3 (6.1)
Number of surgically treated segments	One	34 (68)
	Two or more	16 (32)
Type of surgery	PVP	32 (78.0)
	PKP	9 (22)
Diabetes	No	34 (73.9)
	Yes	12 (26.1)
Rheumatoid arthritis	No	42 (91.3)
	Yes	4 (8.7)
Pneumonia	No	44 (93.6)
	Yes	3 (6.4)
COPD	No	44 (95.7)
	Yes	2 (4.3)
UTI	No	35(76.1)
	Yes	11 (23.9)
Hypertension	No	31 (67.4)
	Yes	15 (32.6)
Obesity	No	42 (91.3)
	Yes	4 (8.7)
Smoking	No	42 (91.3)
	Yes	4 (8.7)
WBC	Continuous	38 (11.7 ± 13.0)
ESR	Continuous	37 (66.7 ± 33.3)
CRP	Continuous	38 (65.3 ± 74.9)
Time interval to infection	Continuous	41 (6.4 ± 14.1)
Paravertebral abscess	No	1 (9.1)
	Yes	10 (90.9)
Pathogens	*Staphylococcus aureus*	16 (34.0)
	*Enterobacter*	4 (8.5)
	*Staphylococcus epidermidis*	3 (6.4)
	*Roseomonas mucosa*	1 (2.1)
	*Aeromonas hydrophila*	1 (2.1)
	*Acinetobacter*	1 (2.1)
	*Hemolytic streptococcus*	2 (4.3)
	*Enterococcus faecalis*	4 (8.5)
	*Methicillin-resistant staphylococcus aureus*	1 (2.1)
	*Methicillin sensitive staphylococcus aureus*	1 (2.1)
	*Achromobacter xylosoxidans*	1 (2.1)
	*Salmonella*	1 (2.1)
	*Peptpstreptococcus*	1 (2.1)
	*Propionibacterium*	1 (2.1)
	*Salmonela choleraesuiss*	1 (2.1)
	*Coagulase-negative staphylococcal*	2 (4.3)
	*Streptococcus agalactiae*	1 (2.1)
	*Staphylococcus saccharolyticus*	1 (2.1)
	*Parvimonas micra*	1 (2.1)
	*Granulicatella adiacens*	1 (2.1)
	*Serratia marcescens, Stenotrophomonas maltophilia* and *Burkholderia cepacia*	1 (2.1)
	*Corynebacterium* and *Propionibacterium*	1 (2.1)
Outcomes	Recovery	30 (62.5)
	Death	10 (20.8)
	Walking assistance	8 (16.7)

NTS, nontuberculous spondylitis; PVP, percutaneous vertebroplasty; PKP, percutaneous kyphoplasty; COPD, chronic obstructive pulmonary diseases; WBC, white blood cell; ESR, erythrocyte sedimentation rate; CRP, C-reactive protein; UTI, urinary tract infection.

In the TS group, the average time interval from index surgery to the diagnosis of spondylitis was 8.45 ± 11.68 months. All patients received anti-tuberculosis drug treatment after surgery. Among them, one was treated with triple drugs (isoniazid, rifampicin and ethambutol), 13 were treated with quadruple drugs (isoniazid, rifampicin, pyrazinamide and ethambutol), and the remaining 9 were treated with anti-tuberculosis regimens that were not described. Twenty patients underwent revision surgery, among which 2 patients underwent anterior debridement and bone graft fusion; 12 patients underwent combined anterior and posterior debridement, instrumentation, and bone graft fusion; and 6 patients underwent one-stage posterior debridement, fixation and bone graft fusion. The remaining 3 patients were treated by unreported types of surgery. *Mycobacterium tuberculosis* was detected by polymerase chain reaction of the infected tissues in 2 patients, while TS was confirmed by findings from both polymerase chain reaction and acid-fast staining of the infected lesions in 14 patients. In 6 patients, TS diagnosis was made based on histopathological examination of the infected tissues showing granulomatous inflammation and/or caseous necrosis and/or multinucleated giant cells. The remaining 1 case had an unknown method of diagnosis. The average follow-up time was 26.2 ± 25.5 months. At the last follow-up, 11 patients experienced a good recovery (“good” was defined as walking normally without the aid of a walking aid), 8 patients required walking assistance, and 3 patients died (one due to paraplegia, the other due to bacteremia and multiple organ failure, and the third patient did not specify the cause of death).

In the NTS group, the average time interval from the index surgery to the diagnosis of spinal infection was 6.36 ± 14.14 months. All patients received anti-infective treatment after surgery, and there were differences in the use of drugs across the studies. Forty-three patients underwent revision surgery, of whom 8 received anterior debridement and bone graft fusion, 25 received combined anterior and posterior debridement, fixation, and bone graft fusion, and 10 received one-stage posterior debridement, instrumentation and bone graft fusion. The remaining 2 cases were treated with an unknown type of surgery. The growth of pathogenic bacteria was detected in the tissue culture of the lesions for all patients. The average follow-up time was 16.7 ± 12.1 months. At the end of the follow-up, 30 patients had a good recovery, 8 patients required walking assistance, and 10 patients died.

### Comparison of clinical features between the TS and NTS groups

The comparison results of the clinical characteristics of patients in the TS group and the NTS group are shown in Table 3. The analysis results showed that patients in the TS group were more likely to have a history of trauma before PVP/PKP treatment. However, due to the small number of TS groups providing trauma history data, this result may be biased. Analyzing the characteristics of infection after its occurrence, we found that the infection time of the TS group patients was longer than that of the NTS patients, while the ESR index of the NTS group patients was higher than that of the TS group patients, but these differences were not statistically significant. There were no significant differences between the TS group and the NTS group in other clinical data.

**Table 3:** Comparison of clinical features between TS and NTS group.

Variable	Categories	TS (*n*)	NTS (*n*)	Statistics	*P*-value
Age (years)	Continuous	23 (71.5 ± 8.7)	50 (70.5 ± 10.4)	0.442	0.695
Sex	Female	19	32	2.591	0.107
	Male	4	18		
Preoperative neurological dysfunction	No	10	10	–	1.000
	Yes	1	0		
Trauma	No	6	21	4.359	**0**.**037**
	Yes	8	5		
Location	Thoracic	7	15	0.836	0.658
	Lumber	15	21		
	Thoracic and Lumber	1	3		
Number of surgically treated segments	One	17	34	0.262	0.609
	Two or more	6	16		
Type of surgery	PVP	15	32	1.244	0.265
	PKP	8	9		
Diabetes	No	15	34	0.243	0.622
	Yes	7	12		
Rheumatoid arthritis	No	21	42	-	1.000
	Yes	1	4		
COPD	No	20	44	-	0.319
	Yes	2	2		
Hypertension	No	15	31	0.004	0.948
	Yes	7	15		
WBC	Continuous	17 (7.4 ± 2.3)	38 (11.7 ± 13.0)	2.130	0.186
ESR	Continuous	17 (52.4 ± 19.7)	37 (66.7 ± 33.3)	7.612	0.052
CRP	Continuous	17 (42.7 ± 34.4)	38 (65.3 ± 74.9)	5.677	0.130
Time interval to infection	Continuous	23 (8.5 ± 11.7)	41 (6.4 ± 14.1)	0.423	0.549
Paravertebral abscess	No	1	1	-	1.000
	Yes	9	10		

Bold values indicate *P* < 0.05; TS, tuberculous spondylitis; NTS, nontuberculous spondylitis; PVP, percutaneous vertebroplasty; PKP, percutaneous kyphoplasty; COPD, chronic obstructive pulmonary disease; WBC, white blood cell; ESR, erythrocyte sedimentation rate; CRP, C-reactive protein.

### Univariate analyses of risk factors for TS or NTS after PVP/PKP

A comparison of the clinical data between the TS group and the control group is shown in Table 4. Our analysis revealed that TS patients were more likely to have pulmonary tuberculosis and diabetes before receiving PVP/PKP. No significant differences were observed for other clinical characteristics between the two groups.

**Table 4:** Comparison of clinical features between TS and Control group.

Variable	Categories	TS (*n*)	Control (*n*)	Statistics	*P*-value
Age (years)	Continuous	23 (71.5 ± 8.7)	114 (70.5 ± 9.6)	0.828	0.645
Sex	Female	19	86	0.550	0.458
	Male	4	28		
Preoperative neurological dysfunction	No	10	103	< 0.001	1.000
	Yes	1	11		
Trauma	No	6	70	0.503	0.478
	Yes	8	44		
Location	Thoracic	7	49	1.411	0.494
	Lumber	15	59		
	Thoracic and Lumber	1	6		
Number of surgically treated segments	One	17	98	0.782	0.376
	Two or more	6	18		
Type of surgery	PVP	15	80	0.221	0.638
	PKP	8	34		
Diabetes	No	15	104	5.297	**0**.**021**
	Yes	7	12		
Rheumatoid arthritis	No	21	103	0.131	0.717
	Yes	1	11		
Pulmonary tuberculosis	No	9	99	16.467	< **0.001**
	Yes	11	15		
COPD	No	20	101	< 0.001	1.000
	Yes	2	13		
Hypertension	No	15	65	0.949	0.330
	Yes	7	49		

Bold values indicate *P* < 0.05; TS, tuberculous spondylitis; PVP, percutaneous vertebroplasty; PKP, percutaneous kyphoplasty; COPD, chronic obstructive pulmonary disease; WBC, white blood cell; ESR, erythrocyte sedimentation rate; CRP, C-reactive protein.

Similarly, the results of a comparison of the clinical features between the NTS and control subgroups are shown in Table 5. These outcomes showed that diabetes and multiple surgical segments (≥2) were significant factors for NTS after PVP/PKP. In addition, obese patients seemed to be more likely to develop NTS after surgery. Moreover, our study also indicated that a history of preoperative trauma and urinary tract infection were closely related to the occurrence of NTS, although the results were not statistically significant. No significant differences were seen between the two groups in terms of other clinical characteristics.

**Table 5:** Comparison of clinical features between NTS and Control group.

Variable	Categories	NTS (*n*)	Control (*n*)	Statistics	*P*-value
Age (years)	Continuous	50 (70.5 ± 10.4)	114 (70.5 ± 9.6)	0.003	0.994
Sex	Female	32	86	2.253	0.133
	Male	18	28		
Preoperative neurological dysfunction	No	11	103	0.272	0.602
	Yes	0	11		
Trauma	No	21	70	3.490	0.062
	Yes	5	44		
Location	Thoracic	15	49	0.458	0.795
	Lumber	31	59		
	Thoracic and Lumber	3	6		
Number of surgically treated segments	One	34	98	5.558	**0**.**018**
	Two or more	16	18		
Type of surgery	PVP	32	80	0.933	0.334
	PKP	9	34		
Diabetes	No	34	104	6.224	**0**.**013**
	Yes	12	12		
Rheumatoid arthritis	No	42	103	< 0.001	1.000
	Yes	4	11		
Pneumonia	No	44	100	0.619	0.432
	Yes	3	14		
COPD	No	44	101	1.180	0.277
	Yes	2	13		
UTI	No	35	100	3.346	0.067
	Yes	11	14		
Hypertension	No	31	65	1.470	0.225
	Yes	15	49		
Obesity	No	42	112	-	0.057
	Yes	4	12		
Smoking	No	42	111	-	0.106
	Yes	4	3		

Bold values indicate *P* < 0.05; NTS, nontuberculous spondylitis; PVP, percutaneous vertebroplasty; PKP, percutaneous kyphoplasty; COPD, chronic obstructive pulmonary disease; WBC, white blood cell; ESR, erythrocyte sedimentation rate; CRP, C-reactive protein; UTI, urinary tract infection.

### Multivariate logistic analyses of risk factors for TS or NTS after PVP/PKP

A multivariate logistic regression model showed that a history of pulmonary tuberculosis and diabetes were independent risk factors for TS after PVP/PKP (Table 6).

**Table 6:** Multivariate logistic analyses of risk factors for TS after PVP/PKP.

Factors	Categories	Multivariate analysis	
		*P*-value	HR (95% CI)
Diabetes	No	**0.005**	0.165 (0.047–0.580)
	Yes		
Pulmonary tuberculosis	No	**< 0.001**	0.103 (0.034–0.318)
	Yes		

Bold values indicate *P* < 0.05; TS, tuberculous spondylitis; PVP, percutaneous vertebroplasty; PKP, percutaneous kyphoplasty.

Similarly, multivariate analysis found that diabetes and the number of surgical segments were independently associated with the occurrence of postoperative NTS, while urinary tract infection, obesity and a history of trauma did not affect NTS development (Table 7).

**Table 7:** Multivariate logistic analyses of risk factors for NTS after PVP/PKP.

Factors	Categories	Multivariate analysis	
		*P*-value	HR (95% CI)
Diabetes	No	**0.041**	0.301 (0.095–0.954)
	Yes		
Obesity	No	0.783	0.671 (0.039–11.429)
	Yes		
Number of surgically treated segments	One	**0.040**	0.345 (0.125–0.951)
	Two or more		
Trauma	No	0.094	2.771 (0.842–8.722)
	Yes		
UTI	No	0.635	1.420 (0.334–6.046)
	Yes		

Bold values indicate *P* < 0.05; NTS, nontuberculous spondylitis; PVP, percutaneous vertebroplasty; PKP, percutaneous kyphoplasty; UTI, urinary tract infection.

## Discussion

In this study, we summarized the influencing factors of spinal infection after PVP/PKP and analyzed the differences in clinical characteristics between TS and NTS patients. We found that a history of pulmonary tuberculosis and diabetes were closely related to the development of postoperative TS, while diabetes and the number of segments treated with surgery independently affected the occurrence of NTS after vertebral augmentation. Moreover, it appeared that patients with trauma were more likely to develop TS after surgery. These data provide a comprehensive understanding of the factors associated with spondylitis after PVP/PKP and may be helpful for guiding preoperative risk stratification and prevention to reduce or even avoid the occurrence of postoperative spondylitis following PVP/PKP treatment.

Currently, there is still a lack of reports on factors affecting spinal infection after PVP/PKP. Our study found that diabetes was an independent risk factor for TS and NTS after PVP/PKP, similar to previous reports showing that diabetes is an important factor for postoperative spinal infection (35–37), which can significantly increase the risk of a spinal infection caused by several specific bacteria (such as *Staphylococcus aureus* and *Mycobacterium tuberculosis*) (38, 39). The mechanism by which diabetes could increase the incidence of postoperative spinal infection remains unclear. Previous studies have revealed that elevated resistin levels in diabetic patients can impair the chemotaxis and phagocytosis of neutrophils by interfering with phosphatidylinositol-3-kinase-dependent downstream pathways (40, 41). In addition, studies have pointed out that high blood sugar levels can weaken the function of antigen-presenting cells, thereby damaging the adaptive immune response mediated by T cells (7, 42, 43). Furthermore, OVCF patients receiving PVP/PKP treatment are generally of an advanced age and have a relatively low immunity (44, 45). These findings may provide a theoretical explanation for how diabetes can promote the incidence of spinal infection after vertebral augmentation. These data also highlight the importance of insulin use during the perioperative period for diabetes patients. However, it should be noted that whether the use of insulin alone can effectively reduce the presence of postoperative spondylitis in diabetic patients after PVP/PKP deserves further investigation, considering that diabetes is linked to various metabolic disorders (such as dyslipidemia, high uric acid, and hypertension).

Published data suggest that pulmonary tuberculosis is closely associated with the occurrence of TS after PVP/PKP. Although the precise mechanism is unknown, researchers consider that TS can occur in the case of active pulmonary tuberculosis by direct hematogenous dissemination of *Mycobacterium tuberculosis* or indirect spread of this pathogen through proximal para-aortic lymph nodes to the surgical site (8, 9). In contrast, some studies have shown that vertebral augmentation may cause tuberculosis infection by reactivating static *Mycobacterium tuberculosis* or inducing the release of this mycobacterium from infected macrophages to the surgically treated area under the condition of inactive pulmonary tuberculosis (7–9). In addition, for patients with diabetes or any other immunosuppressive disorders, the impaired adaptive immune response may also reactivate *Mycobacterium tuberculosis* or aggravate any existing tuberculosis (7, 42, 43). In this study, we found that pulmonary tuberculosis was a significant predictor for TS after PVP/PKP. This result provides the first statistical evidence to support the above speculations (7–9). Additionally, this finding also emphasizes the importance of monitoring patients with preoperative pulmonary tuberculosis after PVP/PKP, given that the risk of postoperative TS is high in these patients and that TS may progress rapidly in this situation (9).

In addition, this study also showed that the number of segments treated by PVP/PKP was a significant factor associated with NTS after surgery. This is not difficult to understand because more surgical levels are usually correlated with a longer operation time, which is generally considered to increase the risk of infection after spinal surgery (36). Another possible explanation may be the fact that more surgical segments commonly reflect severe preoperative trauma, which can likely reduce the specific adaptive immunity of T cells (46–48), thus leading to postoperative spondylitis. Another finding of this study was that obesity might increase the incidence of postoperative spondylitis after PVP/PKP, consistent with the findings of previous observations (49). A possible reason is that the abnormal regulation of hormones and adipokines in obese patients likely compromises T-cell function, thereby weakening the adaptive immune response to infection in this population (50).

Interestingly, our analysis found that preexisting infection in other regions of the body did not contribute to NTS occurrence after PVP/PKP, which contradicts previous reports in the literature (2, 5, 6). Prior data have indicated that vertebral augmentation as an invasive operation may lead to the relative susceptibility of the surgical area (the principle of *locus minoris resistentia*) (2), which then creates a microenvironment suitable for pathogens to invade the surgical site, thereby resulting in subsequent infection. Moreover, during the process of bone cement infusion, repeated C-arm fluoroscopy and frequent personnel movements during the surgery may also increase the risk of postoperative spinal infection. These factors offer a possible route for the development of spondylitis caused by external pathogens after PVP/PKP therapy, although this idea requires further confirmation.

In addition, frailty is one of the most serious global public health challenges we face right now. Rapidly aging populations have brought about an increase in the number of frailty older adults, which in turn has put increasing pressure on healthcare systems worldwide (51, 52). When a stressful event (e.g., acute illness, trauma) occurs, the functional capacity of frailty individuals deteriorates rapidly, but the patients we included did not have a complete frailty evaluation, so “frailty” was not included in this study, but its It is still a very meaningful variable that deserves further exploration in the future (53).

### Limitations

Most of the included studies failed to provide complete clinical data of the patients, which may introduce bias into the results. However, to minimize the heterogeneity among studies and to make the analysis results more reliable and the statistical analysis feasible, we simplified the grouping criteria for most variables in the data processing.

## Conclusion

The present study performed a comprehensive summary of the risk factors for vertebral infection after PVP/PKP. We found that a history of pulmonary tuberculosis and diabetes were independent risk factors for TS. For NTS, our analysis revealed that diabetes and the number of surgically treated segments significantly influenced the occurrence of postoperative spinal infection. These data may be helpful for guiding risk stratification and preoperative prevention for patients, thereby reducing the incidence of vertebral osteomyelitis after PVP/PKP.

## Data Availability

The raw data supporting the conclusions of this article will be made available by the authors, without undue reservation.
