# Corrigendum: Risk factors for tuberculous or nontuberculous spondylitis after percutaneous vertebroplasty or kyphoplasty in patients with osteoporotic vertebral compression fracture: A case-control study

**DOI:** 10.3389/fsurg.2022.1044417

**Published:** 2022-10-05

**Authors:** Bo-Wen Zheng, Fu-Sheng Liu, Bo-Yv Zheng, HuaQing Niu, Jing Li, Guo-Hua Lv, Ming-Xiang Zou, Zhun Xu

**Affiliations:** ^1^ Department of Spine Surgery, The First Affiliated Hospital, Hengyang Medical School, University of South China, Hengyang, China; ^2^ Department of Spine Surgery, The Second Xiangya Hospital, Central South University, Changsha, China; ^3^ Musculoskeletal Tumor Center, Peking University People’s Hospital, Peking University, Beijing, China; ^4^ Department of Orthopedics Surgery, General Hospital of the Central Theater Command, Wuhan, China

**Keywords:** percutaneous vertebroplasty, percutaneous kyphoplasty, tuberculous spondylitis, nontuberculous spondylitis, pyogenic spondylitis, risk factors

A corrigendum on Risk factors for tuberculous or nontuberculous spondylitis after percutaneous vertebroplasty or kyphoplasty in patients with osteoporotic vertebral compression fracture: A case-control study by Zheng B-W, Liu F-S, Zheng B-Y, Niu HQ, Li J, Lv G-H, Zou1 M-X and Xu Z. (2022) Front. Surg. 9: 962425. doi: 10.3389/fsurg.2022.962425

**Incorrect Funding**

In the published article, there was an error in the Funding statement. [This work was supported by the National Natural Science Foundation of China (81871821 to JL and 82002364 to MXZ) and Project for Clinical Research of Hunan Provincial Health Commission (20201956 to MXZ and Natural Science Foundation of Hunan Province)]. The correct Funding statement appears below.

**Funding**

[This work was supported by the National Natural Science Foundation of China (81871821 to JL and 82002364 to MXZ), Natural Science Foundation of Hunan Province (2022JJ40403 to ZX), and Project for Clinical Research of Hunan Provincial Health Commission (20201956 to MXZ)]

The authors apologize for this error and state that this does not change the scientific conclusions of the article in any way. The original article has been updated.

